# Profiling Phenolic Composition in Pomegranate Peel From Nine Selected Cultivars Using UHPLC-QTOF-MS and UPLC-QQQ-MS

**DOI:** 10.3389/fnut.2021.807447

**Published:** 2022-01-24

**Authors:** Guowei Man, Lei Xu, Yongtao Wang, Xiaojun Liao, Zhenzhen Xu

**Affiliations:** ^1^College of Food Science and Nutritional Engineering, China Agricultural University; Beijing Key Laboratory for Food Non-thermal Processing; Key Lab of Fruit and Vegetable Processing, Ministry of Agriculture and Rural Affairs, Beijing, China; ^2^Institute of Quality Standard & Testing Technology for Agro-Products, Chinese Academy of Agricultural Sciences; Key Laboratory of Agro-food Safety and Quality, Ministry of Agriculture and Rural Affairs, Beijing, China

**Keywords:** pomegranate peel, cultivars, phenolic composition, UHPLC-QTOF-MS, UPLC-QQQ-MS, MRM

## Abstract

Pomegranate is widely cultivated across China, and the phenolics in its peel are principal components associated with health benefits. Ultra-high performance liquid chromatography coupled to a quadrupole time-of-flight mass spectrometer (UHPLC-QTOF-MS) and ultra-performance liquid chromatography coupled to a triple quadrupole mass spectrometer (UPLC-QQQ-MS) were used in this study, aiming at profiling the total phenolic composition in pomegranate peel from nine selected cultivars in 7 production areas. Sixty-four phenolic compounds were identified or annotated, and 23 of them were firstly reported in pomegranate peel. Principal component analysis (PCA) plots show differences and similarities of phenolics among nine cultivars. Furthermore, 15 phenolic compounds were quantified with the standards, and punicalagin, ellagic acid, gallocatechin, punicalin, catechin, and corilagin were found to be dominant. Punicalagin weighed the highest content (28.03–104.14 mg/g). This study can provide a deeper and more detailed insight into the phenolic composition in pomegranate peel and facilitate the health-promoting utilization of phenolics.

## Introduction

Pomegranate (*Punica granatum* L.) has been cultivated with a wide geographical global distribution, namely, China, India, Russia, Iran, Uzbekistan, Afghanistan, Spain, Italy, Greece, Morocco, and America (1). China possesses the genetic diversity of pomegranate cultivars, and pomegranates are planted in 7 main production areas (Xinjiang, Yunnan, Sichuan, Shanxi, Henan, Anhui, and Shandong) (2).

Pomegranate peel is about 50% proportion of the fruit weight. Compared with any other anatomical part of the fruit, pomegranate peel has the most promising pool of phenolics (3). With medicinal and nutritional benefits, a variety of phenolic compounds (such as phenolic acids, flavonoids, and tannins) have attracted the attention of many researchers, and many food products and supplements (such as stabilizers, preservatives, prebiotics, and quality enhancers) have been developed based on pomegranate peel (4). Phenolic compounds, as secondary metabolites, are inhomogeneously distributed in plants, and there is a close relationship between phenolic structures and properties (5). In addition, the content of phenolics may have a significant effect on related health benefits, which lead researchers to extract, separate, and quantify these compounds with rapid, simple, environmentally friendly, and comprehensive methods (6). Thus, more effective and comprehensive approaches for annotation and quantification of the phenolic compounds in pomegranate peel were of vital importance.

For qualitative analysis, additional structural information for identification is provided by tandem mass spectrometry (MS/MS or MS^*n*^) experiments (7). With exact mass and highly sensitive and convenient manner of fragmentation pattern, high-resolution and accuracy mass spectral detectors possess the advantages for qualitative studies (8). Ultra-high performance liquid chromatography coupled to a quadrupole time-of-flight mass spectrometer (UHPLC-QTOF-MS) has been used to investigate the composition of food. LC-MS has been widely used for phenolic compound identification in pomegranate peel, and the discovery covers a wide range of phenolic compounds. Also, the comparison among different cultivars is another issue that draws attention from researchers. Abid et al. (9) investigated the phenolic profile of four Tunisian pomegranate peels by liquid chromatographic tandem mass spectrometry (LC-MS/MS) with a total of 24 phenolic compounds differently distributed among them. In addition, the high-resolution instrument of the LC-MS is used. Du et al. (10) investigated phenolic compounds in pomegranate peel from two producing areas in China using the UHPLC-QTOF-MS with the identification of gallic acid, punicalagin, catechin, and ellagic acid. However, there is little comprehensive research regarding the phenolic composition discovery and comparison with different cultivars in pomegranate peel using the UHPLC-QTOF-MS. For quantification, multiple reaction monitoring (MRM), generally requiring standards, becomes a powerful analysis mode in LC-MS/MS and can be used for absolute quantitation of targeted compounds (11, 12). Although MRM has been applied to pomegranate peel investigation (13), over a dozen of main phenolic compounds in pomegranate peel to be absolutely quantified in one analysis under MRM mode are scarcely reported.

Based on the diversity of pomegranate cultivars in China, the aim of this study was to comprehensively investigate the phenolic composition from nine selected cultivars by UHPLC-QTOF-MS and ultra-performance liquid chromatography coupled to a triple quadrupole mass spectrometer (UPLC-QQQ-MS) with optimized conditions under MRM mode. With nine cultivars to be investigated and the use of UHPLC-QTOF-MS, more phenolic compounds are expected to be discovered or understood in this study.

## Materials and Methods

### Main Chemicals and Reagents

Methanol (HPLC grade, 99.9%), ethanol (HPLC grade, 99.8%), acetonitrile (LC/MS grade, 99.9%), and formic acid (LC/MS grade) were all purchased from Thermo Fisher Scientific Technology Co., Ltd. (Shanghai, China). Standard compounds, namely, punicalagin, punicalin, corilagin, ellagic acid, gallic acid, catechin, epicatechin, epicatechin gallate, gallocatechin, epigallocatechin, epigallocatechin gallate, kaempferol-3-O-glucoside, isoquercitrin, luteolin-7-O-glucoside, naringenin-7-O-glucoside, and rutin, were all purchased from Yuanye Biotechnology Co., Ltd. (Shanghai, China).

### Sample Preparation for Chromatographic Analyses

There were seven main production areas in China and we selected the cultivars from all seven production areas with the most representativeness. Nine cultivars of pomegranate fruit, Red agate (RA) from Huaiyuan County (Anhui Province, China), Tunisian Soft Seed (TSS) from Yingyang city (Henan Province, China), Sweet With Green Seed (SGS) from Mengzi County (Yunnan Province, China), Green Peel Soft Seed (GPSS) from Huili County (Sichuan Province, China), Green Peel (GP) from Zaozhuang city (Shandong Province, China), Net Skin Sweet (NSS) from Lintong district (Shanxi Province, China), Piyaman (PYM) from Hetian prefecture (Xinjiang Uygur Autonomous Region, China), Acidic Pomegranate (AP) from Kashgar prefecture (Xinjiang Uygur Autonomous Region, China), Sweet Pomegranate (SP) from Kashgar prefecture (Xinjiang Uygur Autonomous Region, China) were collected. Supplementary Figure 1 (the map was generated by EdrawMax 10.5) shows the overview of geographical distribution with selected cultivars in China. Fresh pomegranates of all cultivars were purchased from their production areas, respectively, and then transported by air to the laboratory. The peel was manually separated, dried by the lyophilization method, and ground by a JYS-M01 grinder (Joyoung Co., Ltd, Jinan city, China). The powder was sieved by a 40 mesh sieve and the undersize was collected. Water is an environmentally friendly solvent and it is widely used in phenolic extraction of pomegranate peel (13, 14). The acidic solution could contribute to the stability of phenolics (15). With some modifications of published articles (13–15), the extraction procedure was carried out with a solid-solvent ratio of 1 g: 50 ml (0.3% formic acid aqueous solution) for half an hour. Subsequently, the extracted solution was filtered by a 0.22 μm polyethersulfone membrane of the water system. The samples (1.5 ml LC vials) were stored at −80°C after extraction until they are run. The experiment was repeated three times. In each repeat, we prepared two samples with one injection for each one.

### Annotation and Identification of Phenolic Compounds

The analysis of phenolic composition among nine cultivars was performed using the UHPLC-QTOF-MS (1290 and 6560, Agilent, Santa Clara, CA, USA) and the method of Xu et al. (16) was referred to with some modifications.

Preliminary separation was performed by UHPLC at the flow rate of 0.4 ml/min with the column (Acquity UPLC HSS T3, 1.8 μm, 2.1 × 150 mm, Waters, Milford, MA, USA) maintained at 40°C and samples maintained at 4°C. Mobile phase A was 0.2% formic acid aqueous solution and B was acetonitrile. The solvent gradient was as followed: 0–11.50 min, 5–30% B; 11.50–11.51 min, 30–100%; 11.51–15.00 min, 100–100% B; 15.00–15.01 min, 100–5%; and 15.01–18.00 min, 5–5% B. The injection volume was 2 μl for all samples.

The MS conditions for positive and negative ion modes were the same in gas temperature (325°C), drying gas flow (7 L/min), spray voltage (35 psi), sheath gas temperature (350°C), sheath gas flow (11 L/min), and fragmentation voltage (380 V). The capillary voltage for positive ion mode was 3,500 V and for negative ion mode was 3,000 V, while the nozzle voltage for positive ion mode was 0 and positive ion mode was 1,500 V, respectively. Data acquisition was performed by TOF MS mode with the *m/z* range of 50–1,200 and the acquisition rate of 2 spectra/s. The quality control (QC) sample, a mixture of aliquots from every sample, was inserted into the queue every 5 samples to ensure the stability and repeatability of the system. Reference ions were 121.050873 and 922.009798 for positive ion mode, and 112.9855 and 1033.9881 for negative ion mode.

The MS/MS was performed under the auto MS/MS mode with QTOF only. The mass acquisition range was 30–1,200 with an acquisition rate of 4 spectra/s. The four highest responding parent ions were selected to be fragmented in every acquisition cycle. Separately, the samples of TSS cultivar were acquired three times under the collision energy of 10, 20, and 40 eV with other conditions the same as those under MS conditions.

### Quantification of Main Phenolic Compounds

The quantification of main phenolic compounds was performed using a UPLC-QQQ-MS (Acquity I Class and Xevo-TQ-S, Waters, Milford, MA, USA).

Chromatographic separation was firstly carried out using a UPLC at the flow rate of 0.3 ml/min with the column (ACQUITY UPLC HSS T3, 1.8 μm, 2.1 × 150 mm, Waters, Milford, MA, USA) maintained at 40°C and samples maintained at 8°C. Mobile phase A was 0.3% formic acid aqueous solution and B was acetonitrile. The solvent gradient was as followed: 0–10.00 min, 5–27.5% B; 10.00–12.50 min, 27.5–55% B; 12.50–13.50 min, 55–100% B; 13.50–16.50 min, 100–100% B; 16.50–16.51 min, 100–5% B; and 16.51–20.00 min, 5–5% B. The injection volume was 2 μl for all samples.

Under both MRM mode and negative ion mode, the optimization for quantification, especially the parameters of cone voltage and collision energy, was performed by both Intellistart and manual tuning with desolvation gas temperature of 500°C, desolvation gas flow of 1,000 L/h, and capillary voltage of 2.0 kV. All standard compounds were dissolved in appropriate solvents (shown in Supplementary Table 1) and diluted by mobile phase A for a series of gradient concentration solutions. Fifteen compounds (punicalagin was the sum of α-punicalagin and β-punicalagin) were targeted quantified with standards. The external standard method was established with the conversion between peak areas and concentrations. The concentration range for the standard curves is shown in Supplementary Table 1 and the *R*^2^ > 0.999.

### Data Processing, Statistical Analysis, and Visualization

Based on the reference of Xu et al. (17) with some modifications, the raw data were first imported to Qualitative analysis b.08.00 software for basic examination. Then the raw data files were converted to abf format by Reifycs Abf converter (RIKEN Center, Japan). The converted files were processed by MS-DIAL 4.24 (RIKEN Center, Japan) with MS/MS information. The MS/MS information was matched with databases, such as the Massbank, GNPs, HMDB, and FooDB with a deviation of 10 ppm for MS and 15 ppm for MS/MS, respectively (18, 19). In addition, fragments of MS/MS in references were also used for annotation. Identification was achieved based on the local standard library with retention time and *m/z*.

After annotation or identification, the compound list with retention time, and the formula was imported into Profinder 10.0 software to extract the peak areas from every sample in MS acquisition. Metaboanalyst (https://www.metaboanalyst.ca/) (20) and SIMCA 14.1 software were used for principal component analysis (PCA).

Statistical analysis was performed by SPSS Statistics 25.0 for the one-way ANOVA, and the bubble plot and the waterfall plot were performed by Origin 2019b.

## Results and Discussion

### Annotation and Identification of Phenolic Compounds

**Figure 1:**
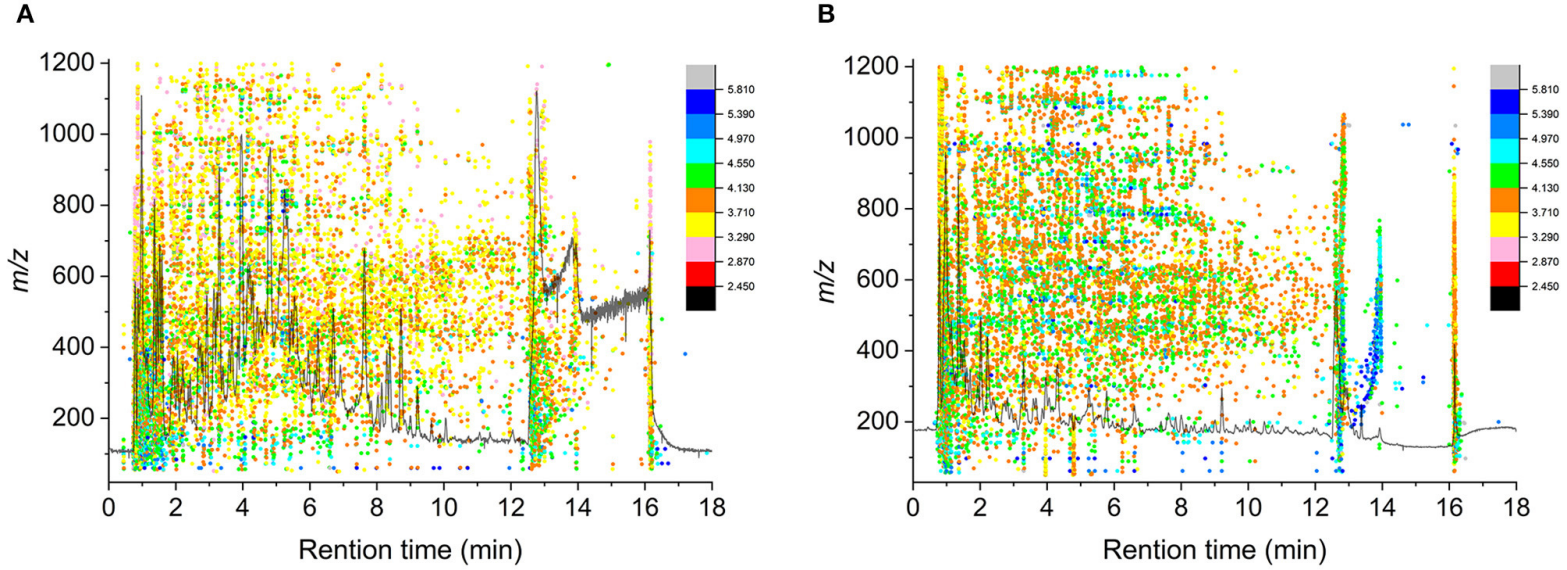
Bubble plot of TIC and acquired data under positive **(A)** and negative **(B)** ion mode.

Figure 1 shows the overview of acquired data under positive (A) and negative (B) ion modes. The total ion chromatogram (TIC) (dark line in the figure) presented the change of summed intensity variation with time. Features were extracted from MS scan data with *m/z*, and every colored bubble, graded by intensity value with log transformed, represented one feature of *m/z* appearing at a specific time. All the colored bubbles distribution in the figure reflected the case of acquired data. Most bubbles were yellow and orange under the positive mode, while they were orange and green or even blue under the negative mode, indicating that the samples had a better response intensity under the negative mode.

**Figure 2:**
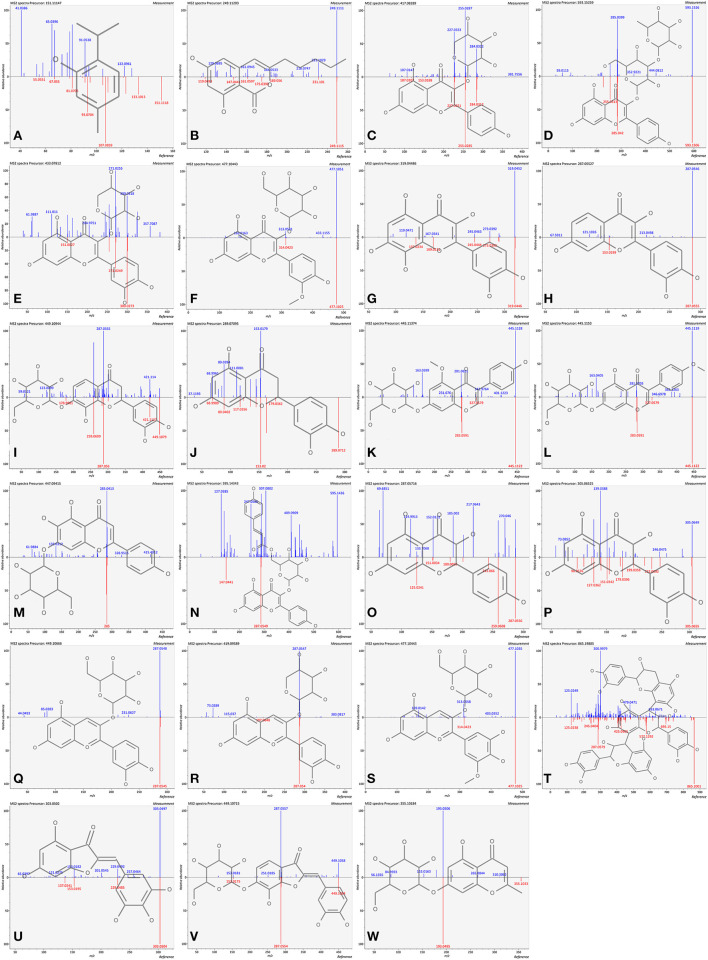
Mirror images of firstly reported compounds in pomegranate peel. **(A)** thymol; **(B)** olivetonide; **(C)** kaempferol-3-O-arabinoside; **(D)** kaempferol-3-glucoside-3″-rhamnoside; **(E)** quercetin-3-O-xyloside; **(F)** isorhamnetin-3-galactoside; **(G)** gossypetin; **(H)** fisetin; **(I)** isookanin-7-O-glucoside; **(J)** eriodictyol; **(K)** isoprunetin-7-O-glucoside, **(L)** biochanin-7-O-glucoside; **(M)** plantaginin; **(N)** tiliroside; **(O)** dihydrokaempferol; **(P)** taxifolin; **(Q)** cyanidin-3-O-galactoside; **(R)** cyanidin-3-O-alpha-arabinoside; **(S)** petunidin 3-galactoside; **(T)** procyanidin C1; **(U)** bracteatin; **(V)** maritimetin-6-O-glucoside; **(W)** undulatoside A.

The analyte is usually fragmented to obtain information beyond the molecule mass, and the following step is to search a database of molecular structures with tandem MS data (21). According to the databases and references, the features were carefully annotated with MS/MS information in this study. Furthermore, in order to have a more reliable confirmation for some dominant compounds, the comparisons of standards with *m/z* and retention time were imported for further identification. Finally, a total of 64 compounds were annotated by UHPLC-QTOF-MS (Table 1), consisting of 10 hydrolyzable tannins, 4 phenolic acids, and 50 flavonoids. Regarding flavonoids, there was a wide range of variety, namely, 6 flavan-3-ols, 13 flavonols, 4 flavanones, 1 dihydrochalcone, 3 isoflavones, 6 flavones, 2 flavanonols, 9 anthocyanins, 3 procyanidins, 2 aurones, and 1 chromone. Among them, 17 of the annotated compounds were identified with the standards. Twenty-three compounds (mirror images are shown in Figure 2), consisting of 2 phenolic acids (thymol and olivetonide), 6 flavonols (kaempferol-3-O-arabinoside, kaempferol-3-glucoside-3″-rhamnoside, quercetin-3-O-xyloside, isorhamnetin-3-galactoside, gossypetin, and fisetin), 2 flavanones (isookanin-7-O-glucoside and eriodictyol), 2 isoflavones (isoprunetin-7-O-glucoside and biochanin-7-O-glucoside), 2 flavones (plantaginin and tiliroside), 2 flavanonols (dihydrokaempferol and taxifolin), 3 anthocyanins (cyanidin-3-O-galactoside, cyanidin-3-O-alpha-arabinoside, and petunidin-3-galactoside), 1 procyanidin (procyanidin C1), 2 aurones (bracteatin and maritimetin-6-O-glucoside), and 1 chromone (undulatoside A) were annotated for the first time in pomegranate peel. In addition, there were 2 compounds that were not detected in all cultivars. Thymol was not detected in TSS, PYM, AP, and SP, while undulatoside A was not detected in GPSS, PYM, AP, and SP. The two compounds possibly possessed the potential to be characteristic markers for cultivar discrimination. All above results highlighted the presence of a wide variety of phenolic compounds in pomegranate peel.

**Table 1:** Annotation and identification of phenolic compounds by UHPLC-QTOF-MS.

**Adduct type**	**Retention time (min)**	***m/z***	**Formula**	**Error (ppm)**	**Compound name**
**10 hydrolyzable tannins**					
[M-H]-	3.938	1083.0591	C_48_H_28_O_30_	−0.2031	α-punicalagin*^a^*
[M-H]-	4.780	1083.0596	C_48_H_28_O_30_	0.2493	β-punicalagin*^a^*
[M-H]-	2.404	781.0538	C_34_H_22_O_22_	1.0627	Punicalin*^a^*
[M-H]-	6.237	633.0743	C_27_H_22_O_18_	1.5796	Corilagin*^a^*
[M-H]-	7.627	951.0769	C_41_H_28_O_27_	2.5235	Granatin B
[M-H]-	7.176	933.0636	C_41_H_26_O_26_	−0.4287	Galloyl-O-punicalin
[M-H]-	1.590	331.0690	C_13_H_16_O_10_	5.7390	Galloyl-glucoside
[M-H]-	9.277	953.0904	C_41_H_30_O_27_	0.2098	Chebulagic acid
[M-H]-	5.203	635.0900	C_27_H_24_O_18_	1.5273	1,3,6-tri-O-galloylglucose
[M-H]-	7.941	787.1028	C_34_H_28_O_22_	3.6590	1,2,3,6-tetragalloylglucose
**4 phenolic acids**
[M-H]-	2.200	169.0145	C_7_H_6_O_5_	1.7750	Gallic acid*^a^*
[M-H]-	8.700	300.9993	C_14_H_6_O_8_	0.9967	Ellagic Acid*^a^*
[M+H]+	7.983	151.1115	C_10_H_14_O	−1.3235	Thymol*^bc^*
[M+H]+	6.524	249.1120	C_14_H_16_O_4_	−0.4014	Olivetonide*^b^*
**6 flavan-3-ols**
[M-H]-	5.247	289.0720	C_15_H_14_O_6_	0.6919	Catechin*^a^*
[M-H]-	6.517	289.0716	C_15_H_14_O_6_	−0.6919	Epicatechin*^a^*
[M-H]-	8.734	441.0828	C_22_H_18_O_10_	0.2267	Epicatechin gallate*^a^*
[M-H]-	3.300	305.0673	C_15_H_14_O_7_	1.9668	Gallocatechin*^a^*
[M-H]-	4.680	305.0670	C_15_H_14_O_7_	0.9834	Epigallocatechin*^a^*
[M-H]-	6.627	457.0783	C_22_H_18_O_11_	1.5315	Epigallocatechin gallate*^a^*
**13 flavonols**
[M-H]-	8.599	609.1466	C_27_H_30_O_16_	0.8208	Rutin*^a^*
[M-H]-	8.988	463.0887	C_21_H_20_O_12_	1.0797	Isoquercitrin*^a^*
[M-H]-	10.046	447.0939	C_21_H_20_O_11_	1.3420	Kaempferol-3-O-glucoside*^a^*
[M-H]-	11.043	417.0829	C_20_H_18_O_10_	0.4555	Kaempferol-3-O-arabinoside*^b^*
[M-H]-	9.604	593.1524	C_27_H_30_O_15_	2.0231	Kaempferol-3-glucoside-3″-rhamnoside*^b^*
[M-H]-	11.023	463.0903	C_21_H_20_O_12_	4.4700	Quercetin-3-O-glucoside
[M-H]-	8.491	477.0683	C_21_H_18_O_13_	1.6140	Quercetin 3-O-glucuronide
[M-H]-	10.427	433.0783	C_20_H_18_O_11_	1.6394	Avicularin
[M-H]-	9.879	433.0781	C_20_H_18_O_11_	1.2007	Quercetin-3-O-xyloside*^b^*
[M-H]-	7.963	477.1050	C_22_H_22_O_12_	2.4733	Isorhamnetin-3-galactoside*^b^*
[M+H]+	7.720	319.0447	C_15_H_10_O_8_	−0.1881	Gossypetin*^b^*
[M+H]+	9.606	287.0549	C_15_H_10_O_6_	−0.3484	Fisetin*^b^*
[M+H]+	8.978	465.1030	C_21_H_20_O_12_	0.4300	Hyperoside
**4 flavanones**
[M-H]-	10.283	433.1142	C_21_H_22_O_10_	0.4618	Naringenin-7-O-glucoside*^a^*
[M-H]-	10.605	449.1089	C_21_H_22_O_11_	−0.0891	Eriodictyol-7-O-glucoside
[M-H]-	6.160	449.1094	C_21_H_22_O_11_	1.1133	Isookanin-7-O-glucoside*^b^*
[M+H]+	10.605	289.0710	C_15_H_12_O_6_	1.0378	Eriodictyol*^b^*
**1 dihydrochalcone**
[M-H]-	11.031	435.1307	C_21_H_24_O_10_	2.1833	Phlorhizin
**3 isoflavones**
[M-H]-	11.031	445.1150	C_22_H_22_O_10_	2.2915	Isoprunetin-7-O-glucoside*^b^*
[M-H]-	10.228	445.1135	C_22_H_22_O_10_	−1.1458	Biochanin-7-O-glucoside*^b^*
[M+H]+	6.596	271.0603	C_15_H_10_O_5_	0.7378	Genistein
**6 flavones**
[M-H]-	9.098	447.0942	C_21_H_20_O_11_	2.0130	Luteolin-7-O-glucoside*^a^*
[M+H]+	10.443	433.1126	C_21_H_20_O_10_	−0.7388	Apigenin-7-O-glucoside
[M-H]-	10.949	447.0942	C_21_H_20_O_11_	1.9012	Plantaginin*^b^*
[M+H]+	10.643	449.1076	C_21_H_20_O_11_	−0.4453	Luteolin 4′-O-glucoside
[M-H]-	12.070	417.0842	C_20_H_18_O_10_	3.5964	Luteolin-3-O-arabinoside
[M+H]+	5.630	595.1434	C_30_H_26_O_13_	−2.0163	Tiliroside*^b^*
**2 flavanonols**
[M-H]-	6.155	287.0572	C_15_H_12_O_6_	3.8320	Dihydrokaempferol*^b^*
[M+H]+	2.166	305.0657	C_15_H_12_O_7_	0.3278	Taxifolin*^b^*
**9 anthocyanins**
[M]+	10.030	287.0552	C_15_H_11_O_6_	0.5225	Cyanidin
[M]+	10.306	449.1079	C_21_H_21_O_11_	0.3117	Cyanidin-3-O-glucoside
[M]+	9.606	449.1077	C_21_H_21_O_11_	−0.2227	Cyanidin-3-O-galactoside*^b^*
[M]+	10.661	419.0977	C_20_H_19_O_10_	0.9306	Cyanidin-3-O-alpha-arabinoside*^b^*
[M]+	3.815	611.1588	C_27_H_31_O_16_	−3.1907	Cyanidin-3,5-di-O-glucoside
[M]+	8.583	465.1027	C_21_H_21_O_12_	−0.3010	Delphinidin 3-glucoside
[M]+	6.151	433.1126	C_21_H_21_O_10_	−0.7388	Pelargonidin-3-O-glucoside
[M]+	4.499	595.1639	C_27_H_31_O_15_	−3.0580	Pelargonidin-3,5-di-beta-D-glucoside
[M-H]-	7.483	477.1038	C_22_H_22_O_12_	0.0419	Petunidin-3-galactoside*^b^*
**3 procyanidins**
[M-H]-	7.291	577.1367	C_30_H_26_O_12_	2.7030	Procyanidin B1
[M-H]-	5.858	577.1352	C_30_H_26_O_12_	0.1733	Procyanidin B2
[M-H]-	5.258	865.1989	C_45_H_38_O_18_	0.4623	Procyanidin C1*^b^*
**2 aurones**
[M+H]+	8.978	303.0501	C_15_H_10_O_7_	0.4950	Bracteatin*^b^*
[M+H]+	5.465	449.1073	C_21_H_20_O_11_	−1.1133	Maritimetin-6-O-glucoside*^b^*
**1 chromone**
[M+H]+	6.757	355.1026	C_16_H_18_O_9_	0.5632	Undulatoside A*^*bc*^*

*UHPLC-QTOF-MS, Ultra-high performance liquid chromatography coupled to a quadrupole time-of-flight mass spectrometer*.

a *Compounds identified by standards*.

b *Compounds firstly reported in pomegranate peel*.

c *Compounds not detected in all cultivars*.

The phenolic composition was one of the popular fields in pomegranate peel investigations, and differences were shown in references for compounds annotation or identification. El-Hadary and Ramadan (22) identified a total of 43 phenolic compounds in pomegranate peel from Egyptian wonderful variety by the comparison of retention time with standards using HPLC. Eight phenolic compounds (gallic acid, catechin, punicalagin, ellagic acid, luteolin-7-O-glucoside, rutin, quercetrin-3-O-glucoside, and apigenin-7-glucoside) were identical with this study. With the absence of molecular weight information, identification performed by HPLC only may come across the problem of co-elution. Moreover, it is hard to build a library of standards covering all detected compounds. MS can measure the mass-to-charge ratio of ions (23), and with HPLC only, it increases a dimension of separation, especially when the compounds are co-eluted. Fischer et al. (24) investigated the Peruvian pomegranate of unknown cultivar purchased from the local market by HPLC-DAD-ESI/MS^*n*^, and 9 anthocyanins and 23 other phenolic compounds were annotated in pomegranate peel. Five anthocyanins (cyanidin-3-O-glucoside, cyanidin-3,5-di-O-glucoside, delphinidin-3-glucoside, pelargonidin-3-O-glucoside, and pelargonidin-3,5-di-beta-D-glucoside) and 5 other phenolic compounds (ellagic acid, granatin B, punicalin, punicalagin, and gallic acid) were identical to this study. Ambigaipalan et al. (25) analyzed the phenolic compounds in pomegranate peel in California by HPLC-DAD-ESI-MS. They showed a total of 79 phenolic compounds with 16 phenolic acids, 12 flavonoids, 35 hydrolyzable tannins, 8 proanthocyanidins, and 8 anthocyanins. The majority of the compounds could only be annotated as derivatives based on characteristic fragments. However, some ESI-MS instruments have relatively low-resolution, and high-resolution instruments are needed for compounds annotation. As a powerful and robust instrument, a QTOF mass spectrometer has rapidly been embraced by the analytical community (26). Abdulla et al. (27) did the qualitative analysis of phenolic compounds in pomegranate peel (Xinjiang Uygur Autonomous Region, China). Samples were treated by macroporous HPD-300 resin, and a total of 50 phenolic compounds (35 hydrolyzable tannins and 15 flavonoids) were detected under negative mode by HPLC-QTOF-MS. The *m/z* could be accurate to four decimal places. Seventeen compounds were identical to this study. However, HPLC separation needed a total of 90 min with a flow rate of 1.2 ml/min. With the purpose of obtaining a faster and more effective method, we chose UHPLC for preliminary separation and shortened the time to 18 min with a flow rate of 0.4 ml/min, which saved time and solvents. Compared with all these references, UHPLC-QTOF-MS was used to investigate the profile of phenolic compounds in pomegranate peel, which could reduce the possibility of co-elution (compared with HPLC only), increase the accuracy of *m/z* and fragments (compared with LC-MS/MS with low resolution), improve the range of detected compounds, strengthen the confirmation of main phenolic compounds with the standards, and save the time and solvents.

It is noteworthy to have an insight into these compounds. Punicalagin and punicalin are ellagitannins (28). For ellagic acid and punicalagin synthesis, gallic acid is a common precursor (29). To some degree, the synthesis of characteristic tannins in pomegranate peel could explain the collectively reported compounds, such as gallic acid and ellagic acid. The differences of flavonoids come from their structural classes, other substitutions and conjugations, and degree of hydroxylation and polymerization (30). These differences contributed to a large number of annotated flavonoids in this study. Procyanidins, the oligomeric compounds, are composed of catechin and epicatechin monomers (31). Anthocyanins are pigments, which make the flowers and fruits of some plants colored orange, pink, red, violet, or blue (32). The common anthocyanins in pomegranate peel, as discussed before, are cyanidin, delphinidin, and pelargonidin in glucoside form, which provide the natural chromogenic substances on the surface of pomegranate fruits.

Two reasons could explain the differences of phenolic compounds in classes from pomegranate peel. One reason was mainly dependent on the characteristics of cultivars cultivated under different natural conditions. The cultivars in this study covered all the main production areas in China, which are various and different from the cultivars in references. Apart from thymol and olivetonide, the other 21 compounds annotated for the first time were all flavonoids in this study. The various derivatives of the initial phenylpropanoid scaffold play important roles in the plant, such as structural integrity, reproduction, UV photoprotection, and internal regulation of plant cell physiology and signaling (33). Nine selected cultivars in this study were planted and grew with changeable climates and complicated geographical environments, and these factors could lead to the facilitation of flavonoid biosynthesis. Another reason came from experiment procedures. Smaoui et al. (34) summarized the scientific literature on the main active phenolic compounds of pomegranate peel identified and quantified by advances in the separation sciences and spectrometry. The extraction solvent, mobile phase, gradient, and instruments are all influential factors in compound detection. To annotate MS/MS spectra from small molecules, the fastest way is the search of tandem mass spectral library and the search results are influenced by parameters, such as mass accuracy, intensity thresholds, acquisition speed, and so on (35). When compared with conventional methods, the results in this study proved that UHPLC-QTOF-MS was a promising instrument for the reliable and comprehensive investigation of phenolic composition in pomegranate peel with high-throughput, high sensitivity, good resolution, and multi-dimensional data acquisition. Furthermore, the results of all annotated and identified phenolic compounds could be used as the database for further investigations. We hypothesized that the nine cultivars and the use of UHPLC-QTOF-MS could help us to discover and understand more phenolic compounds. The results here might indicate their positive effects on the phenolic compounds annotation.

### PCA for the Phenolic Composition of Nine Cultivars

**Figure 3:**
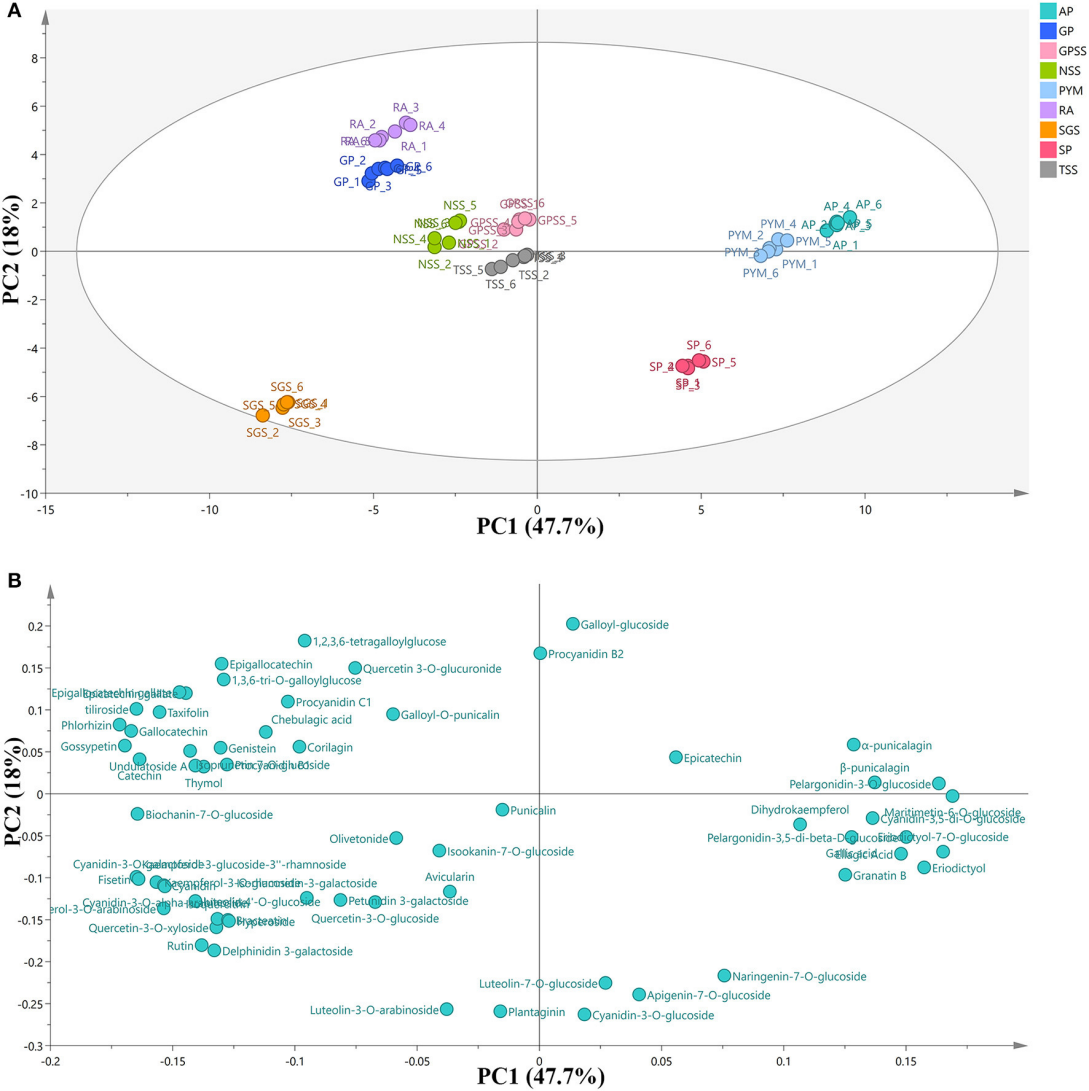
PCA scores plot **(A)** and loading plot **(B)** of phenolic composition in nine cultivars. RA, Red Agate; TSS, Tunisian Soft Seed; SGS, Sweet with Green Seed; GPSS, Green Peel Soft Seed; GP, Green Peel; NSS, Net Skin Sweet; PYM, Piyaman; AP, Acidic Pomegranate; SP, Sweet Pomegranate; PCA, principal component analysis.

Peak areas of the compounds (Table 1) were extracted from MS data with retention time and formula. PCA, a statistical tool, can reduce a large set of variables to a small set with most of the information contained (36). In order to have an overview of the phenolic composition and to compare the differences and similarities among cultivars, PCA is carried out and the results are shown in Figure 3 with its scores plot (A) and loading plot (B). The principal component (PC) represents the percentage of variation. The result of PC1 was 47.7% and PC2 was 18%, indicating that a total of 65.7% of the variation was explained by the two components.

In the PCA score plot, the points in the same color represented the repeated samples for one cultivar. The separations were observed among the cultivars, and the distance among them symbolized the degree of similarity. AP (from Kashgar prefecture) and PYM (from Hetian prefecture) were very close to each other. However, SP, also from Kashgar prefecture, was far from AP or PYM. The three cultivars were all from Xinjiang Uygur Autonomous Region. The results showed that AP and PYM were more similar to each other in phenolic composition, while SP was significantly different from the other two cultivars. RA, GP, GPSS, NSS, and TS were in a big cluster, indicating that these five cultivars were relatively similar in phenolic composition. The unique one was SGS, from Mengzi County, Yunnan Province. It was very far from any other cultivars in the scores plot, showing its specificity in phenolic composition. The relation between cultivars and phenolic compounds was displayed by the combination of the loading plot and the PCA score plot. AP and PYM showed high positive scores along PC1 and the observations in the loading plot indicated that the two cultivars could be positively associated with maritimetin-6-O-glucoside, eriodictyol-7-O-glucoside, pelargonidin-3-O-glucoside, eriodictyol, pelargonidin-3,5-di-beta-D-glucoside, ellagic acid, β-punicalagin, cyanidin-3,5-di-O-glucoside, α-punicalagin, gallic acid, granatin B, and dihydrokaempferol. SGS was highly associated with kaempferol-3-glucoside-3”-rhamnoside, fisetin, kaempferol-3-O-glucoside, kaempferol-3-O-arabinoside, cyanidin-3-O-alpha-arabinoside, luteolin 4'-O-glucoside, hyperoside, bracteatin, quercetin-3-O-xyloside, isoquercitrin, delphinidin 3-galactoside, and rutin. All these phenolic compounds related to SGS were flavonoids and this fact could account for the unique separation in PCA score plot of SGS. In addition, it could be deduced that the cultivar from Yunnan Province had the potential for the use of flavonoids. Li et al. (37) investigated polyphenol composition in pomegranate juices of 10 cultivars from 4 Chinese regions (Kashi prefecture, Zaozhuang city, Mengzi County, Lintong district) with the analysis of environmental factors. The results showed that average temperature and daily temperature difference during maturity and harvest period had big effects on phenolic composition and antioxidant potential. And to some degree, they were also influenced by the latitude and longitude of growing regions. Thus, the differences in phenolic composition of different cultivars in pomegranate peel were also affected by natural conditions. From the PCA plots, we concluded that the separation among the cultivars may be helpful for raw material screening and further purification of specific phenolic compounds.

### Quantification of Main Phenolic Compounds

**Figure 4:**
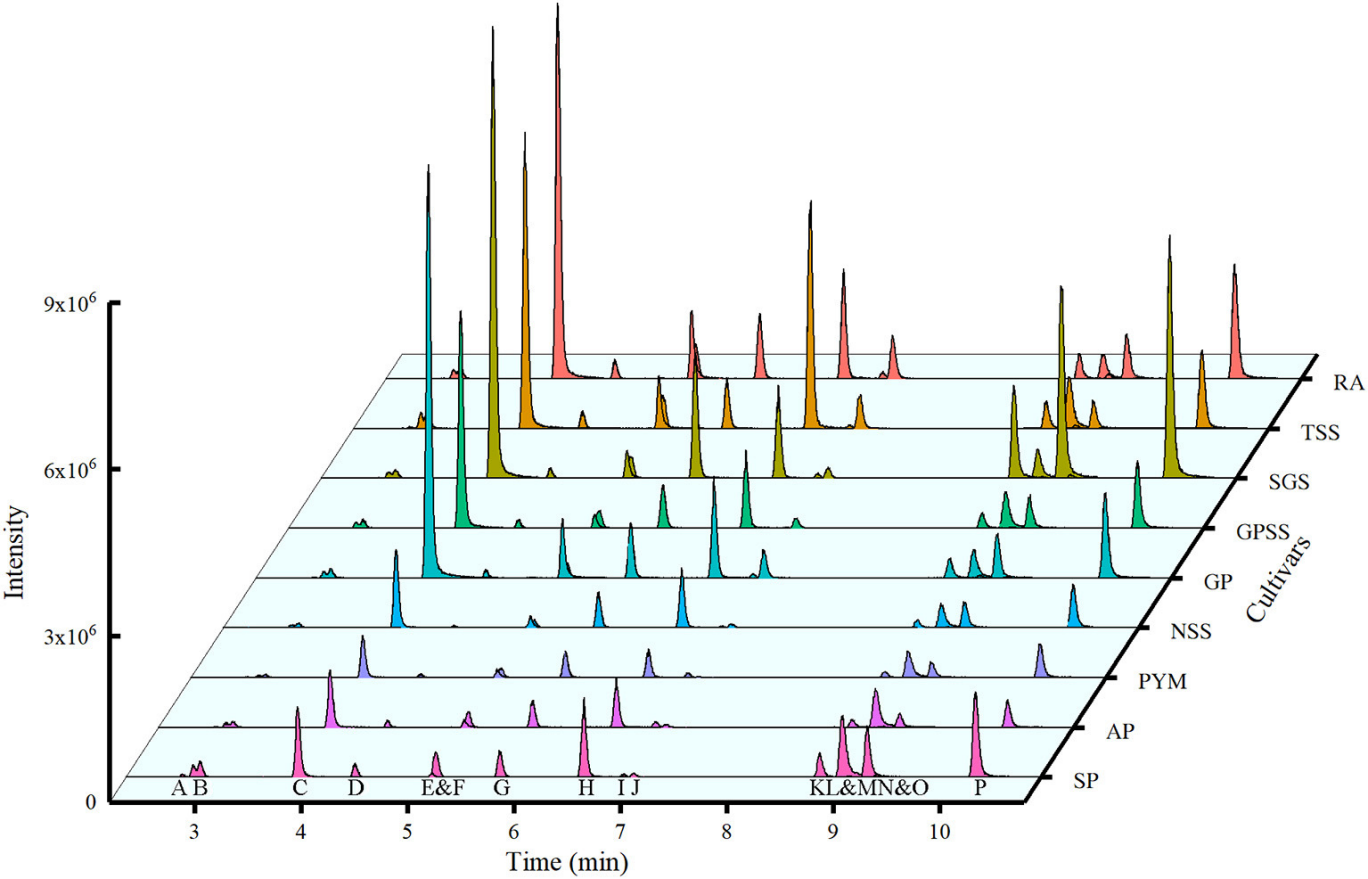
Waterfall chromatogram of ions for quantification from nine cultivars. RA, Red Agate; TSS, Tunisian Soft Seed; SGS, Sweet with Green Seed; GPSS, Green Peel Soft Seed; GP, Green Peel; NSS, Net Skin Sweet; PYM, Piyaman; AP, Acidic Pomegranate; SP, Sweet Pomegranate. The capital letters under the peaks represented the extracted ion chromatography for quantification of the compounds. The name of the compound was A, gallic acid; B, punicalin; C, gallocatechin; D, α-punicalagin; E, epigallocatechin; F, β-punicalagin; G, catechin; H, corilagin; I, epicatechin; J, epigallocatechin gallate; K, rutin; L, ellagic acid; M, epicatechin gallate; N, isoquercitrin; O, luteolin-7-O-glucoside; P, kaempferol-3-O-glucoside.

Fifteen phenolic compounds are quantified by UPLC-QQQ-MS under negative ion mode with the optimized condition of cone voltage and collision energy, as shown in supporting information Table 1. Supplementary Figure 2 showed their mirror images and structures. The waterfall plot (Figure 4) was the extracted ion chromatography for quantification of the compounds under MRM mode.

As shown in Table 2, punicalagin, ellagic acid, gallocatechin, punicalin, catechin, and corilagin dominate, while luteolin-7-O-glucoside is the least among 15 phenolic compounds. Punicalagin (28.03–104.14 mg/g) possessed the highest content among all the compounds in nine selected cultivars, especially in RA and TSS. The highest content of punicalin was obtained in TSS and the least was in PYM. Corilagin content was highest in TSS. SP ranked first with the content of ellagic acid. Catechin and gallocatechin were found with the highest content in SGS.

**Table 2:** Contents of 15 phenolic compounds.

	**RA**	**TSS**	**SGS**	**GPSS**	**GP**	**NSS**	**PYM**	**AP**	**SP**
Punicalagin (mg/g)	100.73 ± 6.25^a^	104.14 ± 11.97^a^	62.63 ± 7.89^c^	62.76 ± 5.58^c^	50.59 ± 6.08^c^	40.03 ± 17.13^d^	28.03 ± 3.52^e^	57.35 ± 4.60^c^	82.19 ± 2.09^b^
Punicalin (μg/g)	524.65 ± 26.18^c^	840.13 ± 105.30^a^	406.82 ± 36.74^d^	437.50 ± 31.79^d^	408.89 ± 35.88^d^	410.24 ± 137.53^d^	202.64 ± 9.84^e^	367.84 ± 21.16^d^	706.63 ± 15.84^b^
Corilagin (μg/g)	191.90 ± 10.09^b^	417.59 ± 54.28^a^	164.03 ± 18.15^bc^	164.50 ± 12.50^bc^	178.65 ± 17.39^b^	203.88 ± 71.86^b^	71.13 ± 4.60^d^	120.67 ± 7.10^c^	154.25 ± 1.29^bc^
Ellagic acid (μg/g)	1737.12 ± 234.30^ef^	3691.85 ± 1053.51^b^	1546.79 ± 127.75^f^	2295.43 ± 139.69^de^	1580.63 ± 147.12^f^	3028.64 ± 290.53^c^	2072.63 ± 96.85^ef^	2738.49 ± 124.08^cd^	4514.06 ± 291.38^a^
Gallic acid (μg/g)	14.03 ± 0.87^de^	40.70 ± 5.82^b^	9.99 ± 1.45^e^	14.18 ± 1.84^de^	13.52 ± 0.82^de^	9.62 ± 3.58^e^	17.59 ± 2.49^d^	21.34 ± 3.10^c^	72.77 ± 2.62^a^
Catechin (μg/g)	300.19 ± 16.89^b^	230.07 ± 34.35^b^	613.45 ± 83.82^a^	234.96 ± 14.82^b^	269.68 ± 26.02^b^	275.82 ± 100.54^b^	115.15 ± 12.44^c^	146.09 ± 11.01^c^	118.37 ± 1.67^c^
Epicatechin (μg/g)	17.24 ± 1.47^b^	9.98 ± 1.27^d^	13.67 ± 1.94^c^	3.82 ± 0.42^e^	12.06 ± 1.14^c^	8.30 ± 2.94^d^	13.59 ± 1.70^c^	20.34 ± 1.51^a^	9.69 ± 0.41^d^
Epicatechin gallate (μg/g)	6.55 ± 0.33^a^	4.40 ± 0.65^b^	2.53 ± 0.37^c^	1.80 ± 0.14^d^	4.26 ± 0.63^b^	2.65 ± 1.02^c^	0.40 ± 0.03^e^	1.61 ± 0.05^d^	1.54 ± 0.14^d^
Gallocatechin (μg/g)	1223.10 ± 72.41^b^	901.40 ± 118.13^c^	1416.54 ± 223.34^a^	805.35 ± 59.18^c^	1428.83 ± 149.09^a^	430.44 ± 200.99^d^	68.61 ± 16.12^e^	163.95 ± 18.13^e^	181.28 ± 6.16^e^
Epigallocatechin (μg/g)	105.77 ± 3.82^a^	77.05 ± 6.41^c^	39.30 ± 4.21^d^	19.27 ± 0.45^f^	88.10 ± 6.73^b^	24.46 ± 6.99^e^	10.36 ± 0.81^g^	10.70 ± 0.91^g^	4.38 ± 0.37^h^
Epigallocatechin gallate (μg/g)	69.80 ± 4.36^a^	59.31 ± 7.59^b^	20.67 ± 2.72^d^	21.48 ± 1.49^d^	49.03 ± 5.93^c^	15.44 ± 5.34^e^	3.79 ± 0.23^f^	8.58 ± 0.30^f^	9.01 ± 0.14^f^
Kaempferol-3-O-glucoside (μg/g)	48.13 ± 2.47^b^	33.96 ± 4.86^c^	99.39 ± 13.05^a^	32.29 ± 2.95^c^	36.97 ± 2.88^c^	33.75 ± 12.60^c^	15.59 ± 1.59^d^	13.55 ± 1.00^d^	40.12 ± 0.76^c^
Isoquercitrin (μg/g)	21.41 ± 1.27^b^	12.52 ± 1.58^cd^	86.11 ± 11.52^a^	17.10 ± 1.37^bc^	20.46 ± 2.05^b^	23.93 ± 9.11^b^	8.49 ± 0.85^d^	8.67 ± 0.71^d^	25.06 ± 0.77^b^
Luteolin-7-O-glucoside (μg/g)	0.25 ± 0.04^e^	0.52 ± 0.07^c^	0.85 ± 0.15^a^	0.14 ± 0.01^f^	0.31 ± 0.02^de^	0.26 ± 0.08^e^	0.25 ± 0.03^e^	0.38 ± 0.04^d^	0.77 ± 0.03^b^
Rutin (μg/g)	21.46 ± 1.65^b^	23.60 ± 3.47^b^	77.22 ± 9.03^a^	14.83 ± 0.97^cd^	18.07 ± 2.00^bc^	13.03 ± 4.54^cd^	6.27 ± 0.55^e^	9.47 ± 0.60^de^	22.02 ± 0.75^b^

*RA, Red Agate; TSS, Tunisian Soft Seed; SGS, Sweet with Green Seed; GPSS, Green Peel Soft Seed; GP, Green Peel; NSS, Net Skin Sweet; PYM, Piyaman; AP, Acidic Pomegranate; SP, Sweet Pomegranate. The letters after each value in the same rows represented the significant difference (p < 0.05) among cultivars and the unit of the content was in dry weight*.

Li et al. (38) investigated 4 main compounds (punicalagin, punicalin, gallic acid, and ellagic acid) in pomegranates from five Chinese cultivars (sweet green-peel pomegranate, sweet red-peel pomegranate, and sour red-peel pomegranate from Huili county, sour red-peel pomegranate from Mengzi County, and sweet Tai-mountain red-peel pomegranate from Taian city) with standards only by HPLC. They showed that the content of punicalagin was much higher than any other phenolic compounds with a range from 61.75 (mg/g DW) to 125.23 (mg/g DW), which was higher than the content of punicalagin in this study. Lu et al. (39) investigated the punicalagin content in pomegranate peel of 14 cultivars collected from seven provinces in China and two cultivars of dried pomegranate peel from drugstores by HPLC. The content ranged from 39.8 to 121.5 mg/g. However, the comparison of the results from the same production areas (provinces) is different between this study and our study. The cultivars, extraction procedure, and the instrument for the experiment could contribute to these differences. It proved that the cultivars in China showed great differences in punicalagin content. Although the differences could come from many aspects, the risk of co-elution should be noticed when the quantification was performed by HPLC only, especially when over a dozen of analytes were analyzed in one analysis. Several overlaps of the retention time were observed among peaks (Figure 4). During the MRM process, the time of MS analysis is focused only on specific masses with all others excluded. Both parent and one or more product ions are monitored simultaneously (40). MRM methods are very suitable to analyze the multiple compounds fastly, sensitively, and simultaneously under the condition of other more abundant compounds presented (41). Chandra et al. (42) provided a method for the quantification of structurally related substances, using the equivalent and molecular weight correction factor. But when comparing the results of Table 2 and Figure 4, we could see that some compounds had high absolute contents with relatively low intensities, such as punicalagin. That was the reason for the necessity to build one-to-one standard curves. However, there were a few reports on the quantification of so many phenolic compounds with standards in pomegranate peel using MRM mode. With the advantages of avoiding the co-elution and quantifying all phenolic compounds in one analysis, the results could be more reliable and accurate under MRM mode with the standards. Therefore, the absolute quantity of 15 phenolic compounds in this study could be helpful for further extraction or by-product development.

## Conclusions

Sixty-four phenolic compounds in pomegranate peel from nine selected cultivars were identified or putatively annotated, and 23 of them were firstly annotated. Thymol and undulatoside A were not detected in all cultivars. Cultivars were well-separated by PCA. For quantification, punicalagin, ellagic acid, gallocatechin, punicalin, catechin, and corilagin dominated among all the phenolic compounds. Punicalagin possessed the highest content with the range from 28.03 to 104.14 mg/g. These results confirmed that the nine cultivars and the use of UHPLC-QTOF-MS were helpful for the discovery and understanding of more phenolic compounds in pomegranate peel. The variety of phenolic compounds revealed the potential of these valuable compounds and the results could be used as the database for pomegranate peel. For absolutely quantified compounds with relatively high contents, more attention should be paid, and further investigations and developments of them are still needed such as extraction, bioactivity, or function.

## Data Availability Statement

The original contributions presented in the study are included in the article/Supplementary Materials, further inquiries can be directed to the corresponding author/s.

## Author Contributions

GM and LX contributed to methodology, investigation, and formal analysis. LX, XL, and ZX contributed to reviewing and editing. GM did data curation and original draft writing. XL helped in supervision and resources. All authors contributed conceptualization to the article and approved the submitted version.

## Conflict of Interest

The authors declare that the research was conducted in the absence of any commercial or financial relationships that could be construed as a potential conflict of interest.

## Publisher's Note

All claims expressed in this article are solely those of the authors and do not necessarily represent those of their affiliated organizations, or those of the publisher, the editors and the reviewers. Any product that may be evaluated in this article, or claim that may be made by its manufacturer, is not guaranteed or endorsed by the publisher.
