# Supplementary material for: Profiling Phenolic Composition in Pomegranate Peel From Nine Selected Cultivars Using UHPLC-QTOF-MS and UPLC-QQQ-MS
Source: Front Nutr. 2022 Jan 24;8:807447. doi: 10.3389/fnut.2021.807447 (PMC8819070; doi:10.3389/fnut.2021.807447)
Supplement: Supplementary file 1 [file Data_Sheet_1.pdf]

Supplementary material

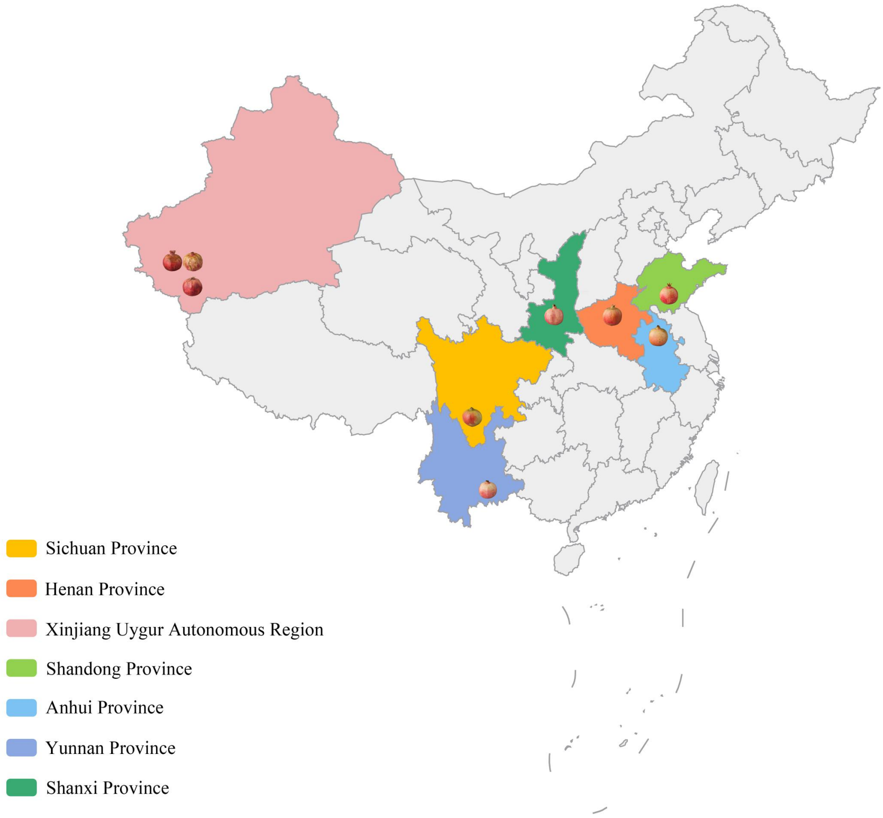

Supplementary Figure 1 The geographical distribution of selected cultivars in China

Supplementary Table 1 Optimization of quantification conditions

Compound name	Molecular formula	Dissolving solvent	Parent ion	Cone voltage （V）	Daughter ion 1	Collision energy 1 (eV)	Daughter ion 2	Collision energy 2 (eV)	Standard concentration (μg/mL)
Punicalagin	$C_{48}H_{28}O_{30}$	Water	1083.55	66	601.22[a]	54	299.08	72	50-2500
Punicalin	$C_{34}H_{22}O_{22}$	Water	781.41	34	601.20[a]	50	299.11	66	1-100
Corilagin	$C_{27}H_{22}O_{18}$	Ethanol	633.37	10	301.05[a]	48	275.03	50	0.5-50
Ellagic acid	$C_{14}H_6O_8$	Methanol	301.23	58	145.01[a]	48	229.06	36	0.4-40
Gallic acid	$C_7H_6O_5$	Ethanol	169.18	6	79.04[a]	24	97.08	26	0.05-5
Catechin	$C_{15}H_{14}O_6$	Ethanol	289.24	8	123.03[a]	38	202.36	22	0.5-50
Epicatechin	$C_{15}H_{14}O_6$	Ethanol	289.24	8	109.04[a]	34	203.06	28	0.025-2.5
Epicatechin gallate	$C_{22}H_{18}O_{10}$	Ethanol	441.31	2	169.07[a]	26	289.16	24	0.005-0.5
Gallocatechin	$C_{15}H_{14}O_7$	Ethanol	305.23	10	125.04[a]	30	179.05	20	1.25-125
Epigallocatechin	$C_{15}H_{14}O_7$	Ethanol	305.23	10	125.04[a]	26	179.05	18	0.05-5
Epigallocatechin gallate	$C_{22}H_{18}O_{11}$	Ethanol	457.37	2	169.07[a]	22	125.03	48	0.05-5
Kaempferol-3-O-glucoside	$C_{21}H_{20}O_{11}$	Ethanol	447.32	70	284.17[a]	36	255.09	54	0.125-12.5
Isoquercitrin	$C_{21}H_{20}O_{12}$	Ethanol	463.32	62	300.20[a]	34	271.12	58	0.1-10
Luteolin-7-O-glucoside	$C_{21}H_{20}O_{11}$	Water and Ethanol	447.324	76	285.08[a]	34	133.10	74	0.00125-0.125
Rutin	$C_{27}H_{30}O_{16}$	Ethanol	609.44	84	300.20[a]	50	271.12	68	0.1-10

[a] The quantification ion for the compound

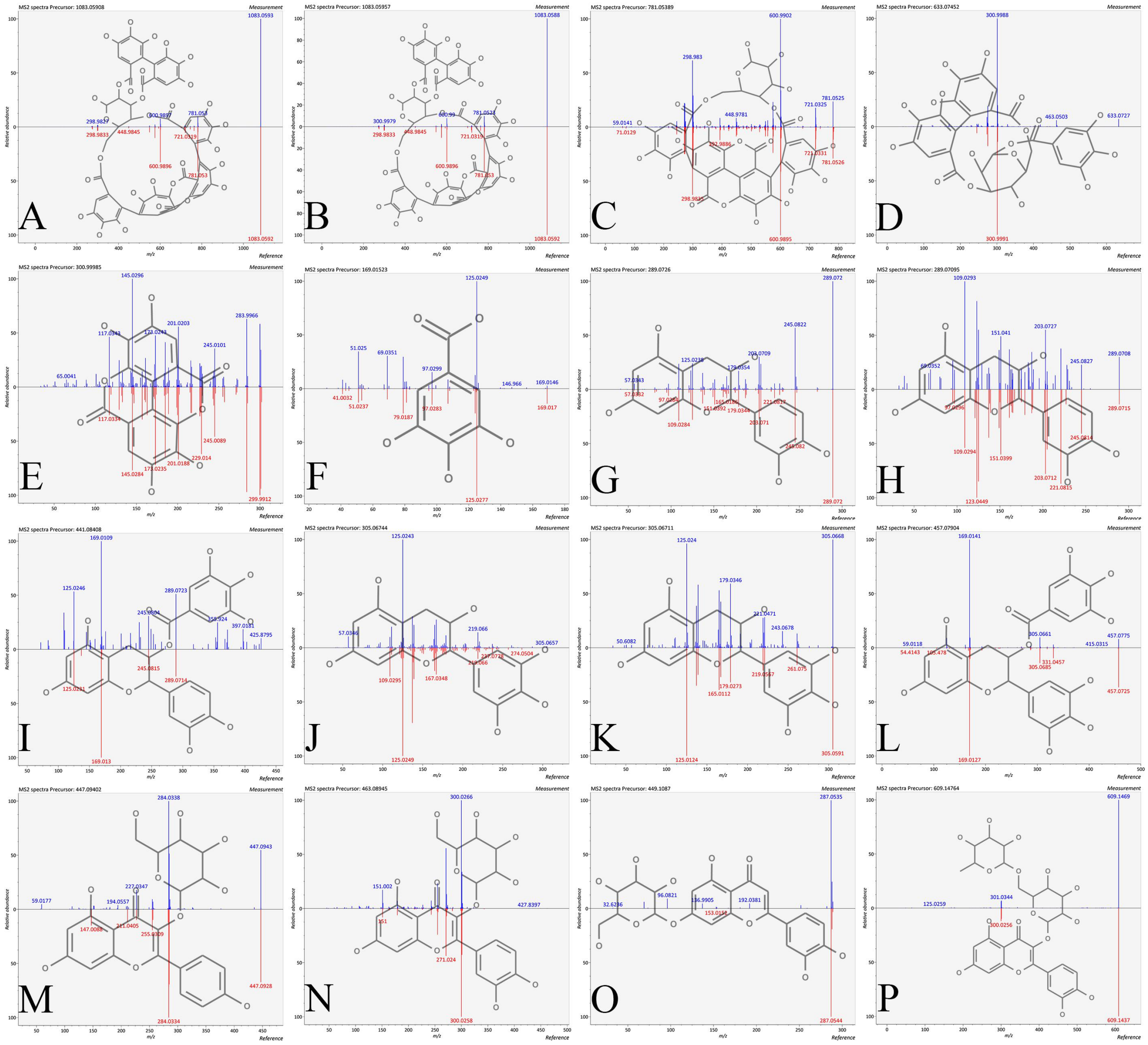

Supplementary Figure 2 Mirror images and structures of quantified compounds

A, α-punicalagin; B, β-punicalagin; C, punicalin; D, corilagin; E, ellagic acid; F, gallic acid; G, catechin; H, epicatechin; I, epicatechin gallate; J, gallocatechin; K, epigallocatechin; L, epigallocatechin gallate; M, kaempferol-3-O-glucoside; N, isoquercitrin; O, luteolin-7-O-glucoside; P, rutin.
